# Dipyridamole Confers Renoprotection Against Rhabdomyolysis-Induced Acute Kidney Injury via Modulation of Oxidative Stress, the PKC/ET-1/PI3K/AKT Signaling Axis, and Apoptosis

**DOI:** 10.3390/jox16060199

**Published:** 2026-10-18

**Authors:** Saleh Alyami, Fatma M. Amin, Maha H. Sharawy

**Affiliations:** Department of Pharmacology and Toxicology, Faculty of Pharmacy, Mansoura University, Mansoura 35516, Egypt

**Keywords:** rhabdomyolysis, AKI, DPM, PKC, apoptosis, ET-1

## Abstract

**Background:** Rhabdomyolysis (RM)-induced acute kidney injury (RIAKI) is a common clinical complication with an increasing incidence. Despite optimal care, the mortality rate is almost 15% due to limited pharmacological interventions. Dipyridamole (DPM), a non-selective phosphodiesterase inhibitor, possesses antioxidant, anti-inflammatory, and antiproliferative properties. This study aimed to investigate the protective effect of DPM against glycerol (GLY)-induced RIAKI, focusing on key AKI-related signaling pathways, including the protein kinase C (PKC)/endothelin-1 (ET-1)/phosphoinositide 3-kinase (PI3K)/protein kinase B (AKT)/mitogen-activated protein kinase (MAPK) cascade, and apoptotic and proliferative responses. **Methods:** Rats were randomly assigned to five groups: control, DPM control, GLY, DPM (10 mg/kg) + GLY, and DPM (20 mg/kg) + GLY. DPM was administered intraperitoneally for 8 days. Notably, the DPM CTRL group received DPM at 20 mg/kg, with no DPM-alone (10 mg/kg) group included. Twenty-four hours later, blood and kidneys were collected. Kidney function, oxidative stress, histopathological changes, and PKC/ET-1/PI3K/AKT/MAPK signaling were evaluated. Additionally, apoptotic markers, Bax and P53, and proliferation-related markers, Ki67 and VEGFA, were assessed immunohistochemically. **Results:** DPM significantly improved kidney function and preserved renal architecture. DPM attenuated oxidative stress, suppressed PKC and ET-1, and modulated the PI3K/AKT/MAPK signaling. Moreover, DPM downregulated Bax and P53 and reduced Ki67 and VEGFA, reflecting attenuation of injury-associated signaling rather than impaired renal repair. **Conclusions:** DPM exerted renoprotective effects against GLY-induced RM-associated AKI through multiple complementary mechanisms.

## 1. Introduction

Acute kidney injury (AKI) is a serious clinical syndrome characterized by a rapid decline in renal function, resulting in impaired renal excretory capacity and disruption in fluid and electrolyte homeostasis [1]. Despite advances in supportive care approaches, patients with AKI have a bleak short-term prognosis, with a high risk of chronic kidney disease (CKD) (8.8-fold), end-stage kidney disease (3.1-fold), and eventual mortality (2.0-fold), compared to non-AKI patients [2,3]. The etiologies of AKI are classified into pre-renal, renal, and post-renal factors, primarily involving ischemia/reperfusion injury (IRI), nephrotoxins, and urinary obstruction [4]. Among the numerous etiologies of AKI, rhabdomyolysis (RM) accounts for approximately 10–50% of cases and represents a major cause of pigment-induced nephropathy [5].

RM is well-defined by the acute destruction of skeletal muscles and muscle fiber apoptosis, followed by the release of muscle cell contents into the blood circulation [6]. Once skeletal muscle contents are in circulation, a well-understood cascade of events occurs, involving the release and potentially lethal accumulation of toxins, including potassium, lactic acid, and predominantly myoglobin, leading to multiple organ failure and most notably AKI. This phenomenon, referred to as RM-induced AKI (RIAKI), is a common complication affecting up to 46% of patients hospitalized and 80% of those requiring intensive care for RM [7,8]. Even with excellent care, mortality is greater than 15%. The incidence of RIAKI has increased 10-fold in the last decade [9].

Although the exact mechanisms of RIAKI remain unclear, its pathophysiology is believed to be triggered by excessive myoglobin accumulation within renal tubules, promoting tubular obstruction, renal endothelial dysfunction and vasoconstriction, oxidative stress, inflammation, apoptosis, and direct nephrotoxicity, ultimately leading to acute renal dysfunction [10,11].

Emerging evidence has identified protein kinase C (PKC) as a therapeutic target in RIAKI [12]. PKC is a subgroup of serine/threonine kinases that, when activated, regulate multiple cellular functions, including cell differentiation, survival, and death. Since tubular apoptosis is a hallmark of AKI, aberrant activation of PKC triggers apoptosis and activates the mitogen-activated protein kinase (p38 MAPK) signaling pathway. Therefore, inhibition of PKC signaling may represent a promising strategy for attenuating renal tubular injury and preserving kidney function.

The glycerol (GLY)-induced RM model is a well-established and extensively used model for studying AKI molecular mechanisms and evaluating potential renoprotective interventions, as it closely recapitulates the clinical features of human RM-associated AKI and the underlying pathological mechanisms involved, including renal vasoconstriction, oxidative stress, tubular obstruction, inflammation, and direct nephrotoxicity [13,14,15].

The antiplatelet dipyridamole (DPM) is a pyridopyrimidine derivative that exerts its effect by inhibiting phosphodiesterase (PDE) nonselectively, with prominent inhibitory activity against PDE3 and PDE5, and blocking uptake of adenosine [16,17]. Beyond its well-established antiplatelet activity, DPM has multiple pharmacological potentials, such as antioxidant, anti-inflammatory, and antiproliferative properties [18,19]. Interestingly, accumulating evidence has highlighted renoprotective potential in both experimental and clinical settings. DPM significantly improved renal function in subtotal nephrectomy rats and was associated with renal vasodilation and decreased podocyte swelling while attenuating the progression of glomerular disease associated with renal mass reduction [20]. Recently, DPM was shown to alleviate necroptosis and renal inflammation via cAMP/CREB signaling pathways in septic AKI mice [21]. Clinically, DPM monotherapy reduced urinary albumin excretion in diabetes patients with normo- or microalbuminuria [22].

However, despite these promising renoprotective properties, the potential therapeutic effect of DPM against RM-induced AKI remains largely unexplored, particularly with respect to the molecular pathways governing oxidative stress, phosphoinositide 3-kinase/protein kinase B/mitogen-activated protein kinases (PI3K/AKT/MAPK) signaling, PKC activation, and apoptosis. Therefore, the current study aimed to investigate the possible renoprotective effects of DPM against glycerol-induced RIAKI in rats and to explore the underlying molecular mechanisms mediating the anticipated protective effects.

## 2. Materials and Methods

### 2.1. Chemicals and Drugs

DPM vial (Dipyridamole, 5 mg/mL, West-Ward Pharmaceuticals Corp., Eatontown, NJ, USA), GLY (AL-Gomhoria Co., Cairo, Egypt), and the other chemicals used in this study were of the finest grade. GLY concentration was adjusted with 0.9% normal saline to 50% *v*/*v* (5 mL GLY was adjusted to 10 mL with 5 mL 0.9% normal saline).

### 2.2. Animals

Thirty 6-week-old male Sprague-Dawley rats weighing 180 ± 20 g were obtained from the Egyptian Organization for Biological Products and Vaccines (VACSERA) in Giza, Egypt. The rats were kept four per cage, under a 12-h light/dark cycle. The study protocol was approved by the Mansoura University Animal Care and Use Committee (MU-ACUC, approval number: PHARM.MS.24.09.105). All procedures strictly adhered to the NIH Guide for the Care and Use of Laboratory Animals (Publication No. 85-23, revised 1985), and the ARRIVE 2.0 guidelines.

### 2.3. Experimental Protocol

In the current study, RIAKI was established in rats using the GLY-induced model. GLY (50% *v*/*v*) was injected intramuscularly (i.m.) at a dose of 10 mL/kg once on day 8 [23]. On the seventh day, all rats were given free access to food for twenty-four hours with water restriction.

Rats were randomized to 5 experimental groups using a computer-generated randomization sequence prior to the start of the experiment. The experimental design was constructed as follows:

1. Control (CTRL) group: rats received 0.9% saline solution for 8 consecutive days.
2. DPM CTRL group: rats were administered DPM (20 mg/kg, i.p.) for 8 consecutive days.
3. GLY group: rats were injected once with 50% *v/v* GLY (10 mL/kg, i.m.) on the eighth day [23].
4. DPM (10 mg/kg) + GLY group: rats received DPM (10 mg/kg, i.p.) [24] for 8 consecutive days. On the eighth day, rats were injected with 50% *v*/*v* GLY (10 mL/kg, i.m.) 1 h after DPM injection.
5. DPM (20 mg/kg) + GLY group: rats received DPM (20 mg/kg, i.p.) [25] for 8 consecutive days. On the eighth day, rats were injected with 50% *v*/*v* GLY (10 mL/kg, i.m.) 1 h after DPM injection.

The study included a DPM 20 mg/kg alone group (DPM CTRL) to assess drug-alone effects in healthy animals. However, the DPM 10 mg/kg-alone group was not included, reflecting an incomplete factorial design.

### 2.4. Specimen Collection

Twenty-four hours after receiving the GLY injection (day 9), the rats were weighed and anesthetized (secobarbital 50 mg/kg, i.p.). Blood samples were collected from the retro-orbital venous plexus in EDTA-free tubes. The sera were separated by centrifugation and used for the immediate assessment of kidney function biomarkers.

**Figure 1:**
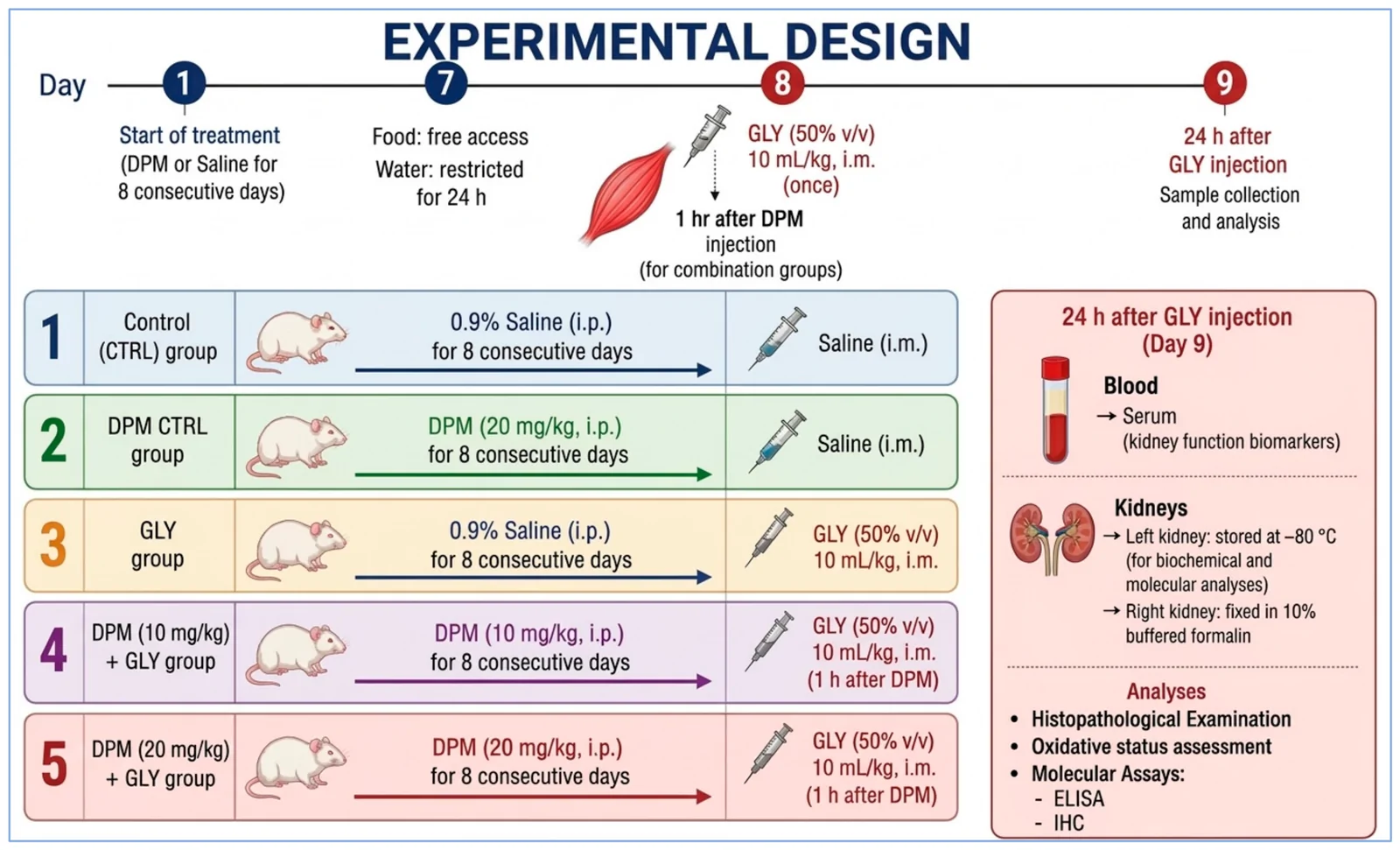
A schematic diagram of the study protocol design. DPM: dipyridamole, GLY: glycerol, i.p.: intraperitoneal injection, i.m.: intramuscular injection, ELISA: enzyme-linked immunosorbent assay, IHC: immunohistochemistry.

The kidneys were isolated and rinsed, and the left kidney was kept at −80 °C for later molecular assay. Phosphate-buffered saline (PBS) was used to homogenize the left kidney. Kidney homogenates were centrifuged at 4000× *g* at 4 °C for 15 min. For additional analysis, the supernatant was stored at −80 °C. The right kidney was preserved for histological and immunological examinations in 10% buffered formalin. To minimize the potential for bias, samples were coded to conceal group allocation; histopathological and IHC quantification (ImageJ analysis, (Fiji ImageJ 1.54p, NIH, Bethesda, MD, USA)) were performed blinded by the pathologist. Figure 1 represents the experimental design of the study.

### 2.5. Serum Biochemical Analysis

In serum, levels of blood urea nitrogen (BUN), serum creatinine, and creatine kinase-MB (CK-MB) were detected using kits from Biomed Diagnostic (Bader City, Egypt). All the procedures were performed according to the instructions provided with the kits.

### 2.6. Estimation of Oxidant/Antioxidant Status

Malondialdehyde (MDA), total antioxidant capacity (TAC), and nitric oxide (NO) were determined in kidney tissue homogenate using commercially available colorimetric kits (Biodiagnostic, Giza, Egypt; Cat. No. MD 25 29, TA25 13, and NO 25 33, respectively).

### 2.7. Determination of Kidney Protein Content of Endothelin 1 (ET1), PKC, MAPK, PI3K, and p-AKT1 Using ELISA

ET1 and PKC were assayed in tissue homogenates using rat ELISA kits (SunLong Biotech Co., LTD., Hangzhou, China, Cat. No. SL0252Ra and SL0608Ra), with detection limits of 0.5 pg/mL and 0.05 ng/mL, respectively. p-AKT (ELK Biotechnology, Wuhan, China, Cat. No. ELK9777), with a detection limit of 6.4 pg/mL, and MAPK and PI3K (MyBiosource, San Diego, CA, USA, Cat. No. MBS267271 and MBS260381, respectively), with detection limits of 0.05 ng/mL and 0.06 ng/mL, respectively, were assayed according to the manufacturers’ instructions.

### 2.8. Histopathological Examination

Kidney specimens were fixed in freshly prepared 10% neutral buffered formalin, processed routinely, and embedded in paraffin. Paraffin sections 5 μm thick were prepared and stained with hematoxylin and eosin (H&E) for histopathological examination. The slides were coded, and each slide was given a specific number according to its corresponding group. However, the pathologist was blinded to these group assignments. The prepared slides from each group were examined under a light microscope (Olympus CH2, Tokyo, Japan). The severity of tubular degeneration, tubular necrosis, fibrosis, and inflammatory cell infiltration was assessed. Renal histopathological alterations were evaluated using a semi-quantitative scoring system as presented in Table 1.

### 2.9. Immunohistochemical (IHC) Evaluation

Paraffin-embedded sections were deparaffinized with xylene and dehydrated in decreasing concentrations of ethanol. Sections were treated with 3% hydrogen peroxide in methanol for 10 min to block endogenous peroxidase activity. For antigen retrieval, tissue sections were processed in EDTA and incubated for 1 h at 37 °C with primary antibodies against Bax (Servicebio, Wuhan, China, Cat. No. GB114122), P53 (Servicebio, Wuhan, China; Cat. No. GB11626), Ki67 (ABclonal Technology, Wuhan, China; Cat. No. A20018), and VEGFA (Bioss Antibodies, Beijing, China; Cat. No. bs-1313R). All procedures were performed at room temperature according to the manufacturer’s instructions using the HRP-DAB detection system. With the help of 3,3′-diaminobenzidine, binding was visualized, and after counterstaining with hematoxylin, slides were examined. Sections were examined using a light microscope with a high-power objective for Bax-, P53-, Ki67-, and VEGF-positive cells. The percentage of positive areas was investigated in 3 slices per group via ImageJ analysis software (Fiji ImageJ 1.54p, NIH, Bethesda, MD, USA).The average percentage was computed.

### 2.10. Statistical Analysis

Statistical analysis was performed using GraphPad Prism 9.0 (GraphPad Software Inc., V 9.0.2, San Diego, CA, USA). Data are presented as mean ± SEM. Parametric data were analyzed using one-way analysis of variance (ANOVA), followed by Tukey’s multiple comparisons post hoc tests. Non-parametric data were analyzed using the Kruskal–Wallis test followed by Dunn’s multiple comparison post hoc test. Statistical significance was confirmed at *p* < 0.05. Tukey’s and Dunn’s tests were used to adjust for within-family multiplicity across the pairwise comparisons.

## 3. Results

Notably, there were no statistically significant differences between the CTRL and DPM CTRL groups in terms of serum kidney function markers, oxidative stress markers, and histopathological findings. Therefore, to simplify the presentation of the results and avoid unnecessary data repetition, the molecular analyses for the DPM CTRL group were limited to PI3K, p-AKT, and MAPK.

### 3.1. Impact of DPM (10 and 20 mg/kg) on Kidney Function Parameters and Serum CK-MB

**Figure 2:**
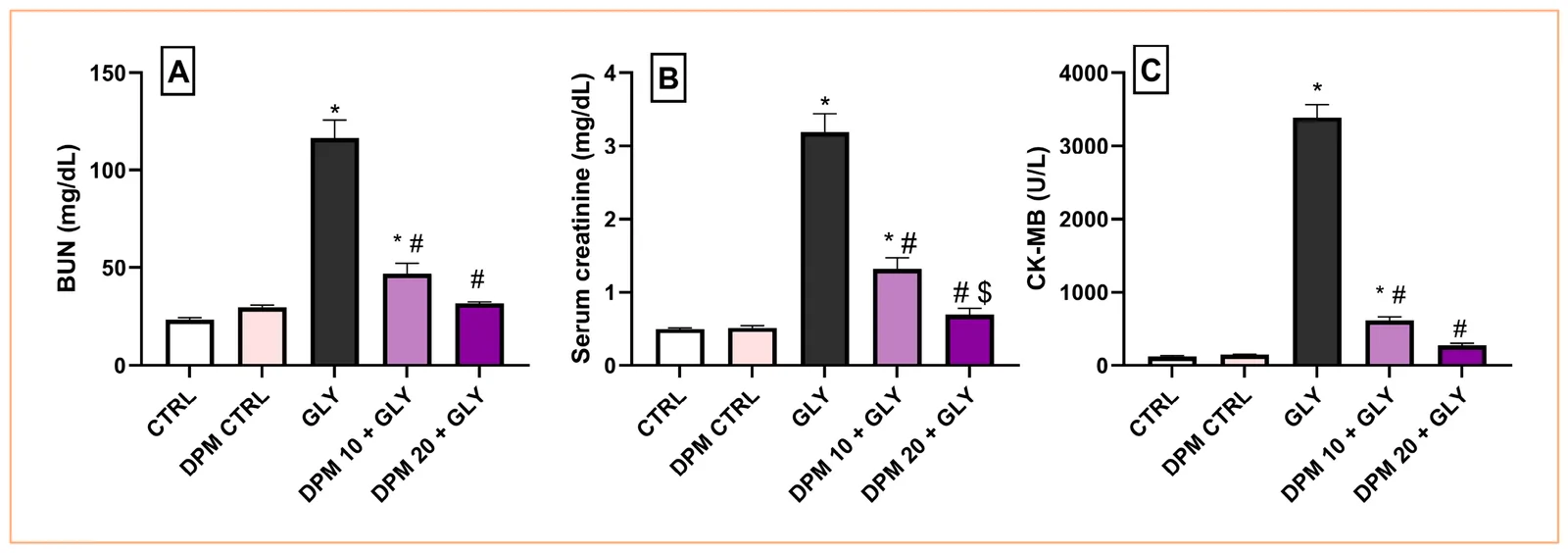
Impact of DPM (10 and 20 mg/kg) on Kidney function parameters. (**A**) BUN; (**B**) Serum creatinine, and (**C**) CK-MB. Values are expressed as mean ± SEM. Data were compared by one-way ANOVA followed by a Tukey–Kramer multiple comparison test (n = 6). *, #, $ indicate *p* < 0.05 compared with CTRL, GLY, and DPM 10 + GLY, respectively. BUN: Blood Urea Nitrogen; CK-MB: creatine kinase-MB; CTRL: control; DPM: Dipyridamole; GLY: glycerol.

Serum BUN, serum creatinine, and CK-MB levels were profoundly elevated in the GLY group, compared with controls (*p* < 0.05). Administration of DPM (10 and 20 mg/kg) produced a significant attenuation in GLY-associated elevated levels of serum BUN, serum creatinine, and CK-MB. Notably, DPM revealed a dose-dependent reduction in serum creatinine, where DPM (20 mg/kg) approached near-control values (Figure 2). Notably, the DPM CTRL group revealed a non-significant difference compared to the CTRL group.

### 3.2. Impact on Histopathological Examination of the Kidney

**Figure 3:**
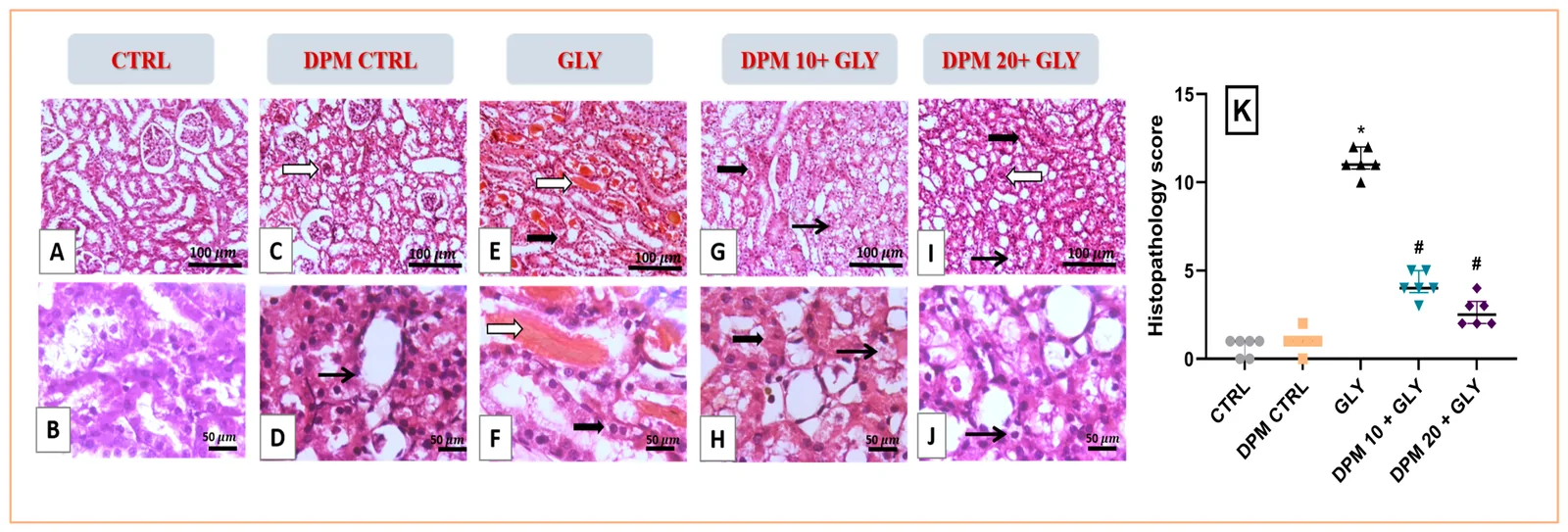
Impact of DPM (10 and 20 mg/kg) on histopathological alterations induced by GLY in kidney tissues of rats. Representative photomicrographs of renal sections from different treatment groups. (**A**,**B**) CTRL, (**C**,**D**) DPM CTRL, (**E**,**F**) GLY, (**G**,**H**) DPM 10 + GLY, (**I**,**J**) DPM 20 + GLY, and (**K**) Semi-quantitative scoring of the histopathological injury. Thin arrow: tubular vacuolation; thick arrow: tubular necrosis; white arrow: intraluminal hyaline cast, desquamated epithelium, or cellular cast; star: calcification. Image magnification: 100×, bar = 100 μm, 400×, bar = 50 μm. Values were expressed as median ± interquartile range. Data were analyzed using the Kruskal–Wallis test followed by Dunn’s multiple comparison test (n = 6). *, # indicate *p* < 0.05 compared with CTRL and GLY, respectively. CTRL: control; DPM: dipyridamole; GLY: glycerol.

The control group shows normal renal parenchymal architecture, displaying preserved architecture in 85–90% of the renal parenchyma, with only occasional tubular pyknosis. The GLY-treated group shows extensive renal injury. Marked tubular necrosis is evident, characterized by shrunken tubular epithelium with pyknotic nuclei (necrosis), or severe tubular swelling with pale cytoplasm (degeneration). Numerous intraluminal eosinophilic hyaline casts admixed with basophilic calcium granules are observed. Additional findings include focal glomerular tuft necrosis, coalescing areas of interstitial fibrosis, and abundant inflammatory infiltrates composed of lymphocytes, plasma cells, and macrophages widely separating necrotic tubules. The DPM-treated group shows diffuse loss of cortical tubular epithelium with either severe tubular necrosis or marked swelling and vacuolation. Most tubules are dilated and contain abundant intraluminal proteinaceous eosinophilic material. The interstitium displays scattered low- to moderate lymphoplasmacytic cell infiltration and fibroblasts (Figure 3). Microscopic Original Images can be found in Supplementary Materials File S1.

### 3.3. Impact of DPM (10 and 20 mg/kg) on Oxidative Status-Related Biomarkers

**Figure 4:**
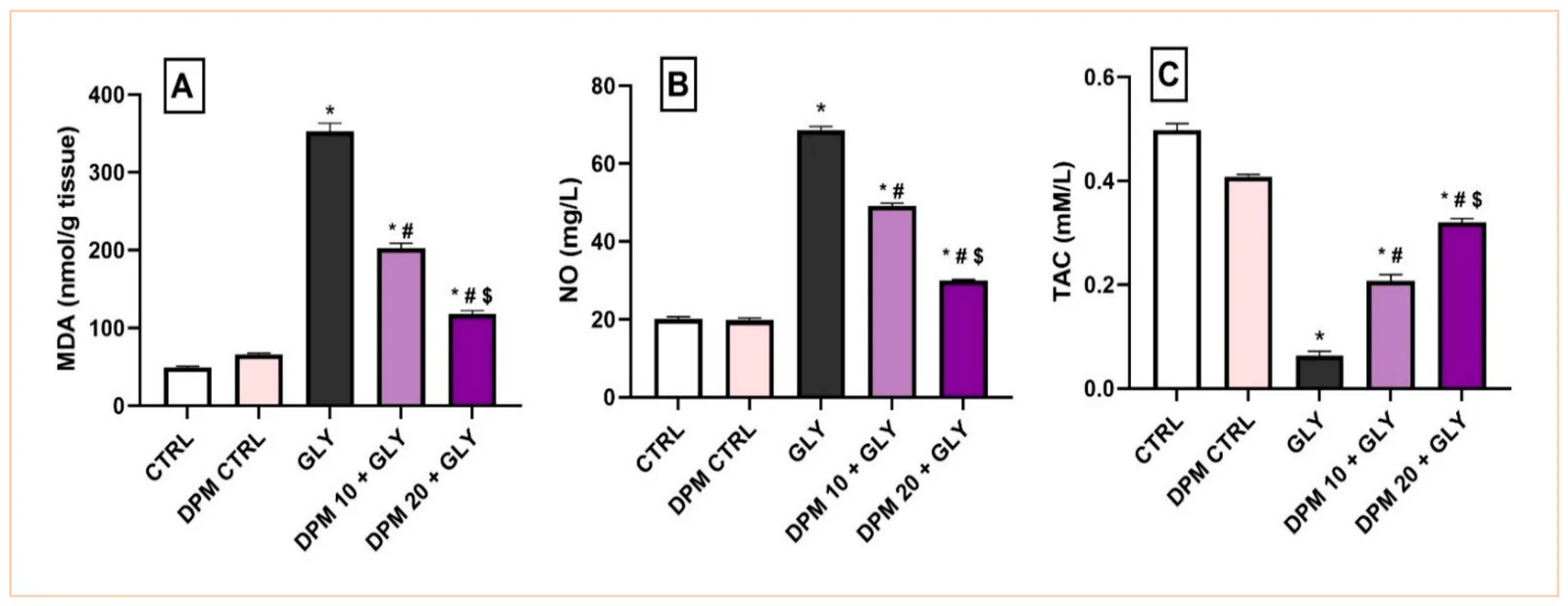
Impact of DPM (10 and 20 mg/kg) on oxidative status-related biomarkers. (**A**) MDA, (**B**) NO, (**C**) TAC. Values are expressed as mean ± SEM. Data were analyzed by one-way ANOVA followed by a Tukey–Kramer multiple comparison test (n = 6). *, #, $ indicate *p* < 0.05 compared with CTRL, GLY, and DPM 10 + GLY, respectively. CTRL: control; DPM: dipyridamole; GLY: glycerol; MDA: malondialdehyde; NO: nitric oxide; TAC: total antioxidant capacity.

As presented in Figure 4, MDA and NO levels were significantly increased in the GLY group, compared with the control group. Notably, the DPM CTRL group revealed a non-significant difference compared to the CTRL group. Treatment with DPM (10 and 20 mg/kg) significantly reduced GLY-induced MDA and NO elevations in a dose-dependent manner. TAC level declined markedly in the GLY group compared with the control. DPM treatment succeeded in restoring TAC levels at both doses (10 and 20 mg/kg), compared to GLY (*p* < 0.05).

### 3.4. Effect of DPM (10 and 20 mg/kg) on Kidney Protein Content of PKC and ET1

**Figure 5:**
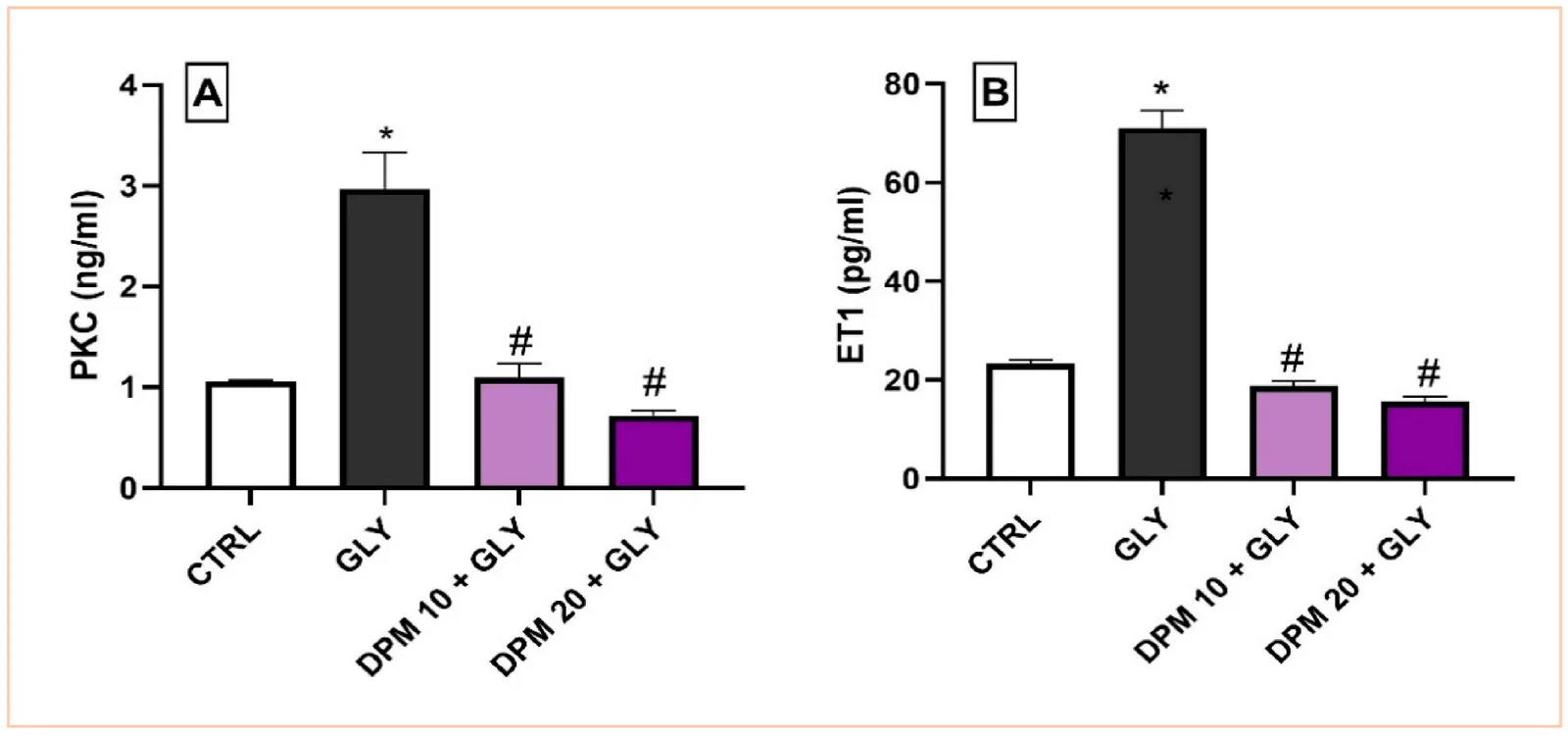
Impact of DPM (10 and 20 mg/kg) on kidney protein content of PKC (**A**) and ET1 (**B**). Values are expressed as mean ± SEM. Data were analyzed by one-way ANOVA followed by a Tukey–Kramer multiple comparison test, n = 4–5 (limited ELISA kit availability). *, # *p* < 0.05 compared with CTRL and GLY, respectively. CTRL: control; DPM: dipyridamole; ET-1: endothelin-1; GLY: glycerol; PKC: protein kinase C.

Kidney levels of PKC and ET1 were significantly increased in the GLY group, compared with the control group. Treatment with DPM (10 and 20 mg/kg) significantly reduced GLY-induced PKC and ET1 elevation compared to the GLY group (*p* < 0.05) Figure 5.

### 3.5. Impact of DPM (10 and 20 mg/kg) on PI3K, p-AKT, and MAPK Signaling Axis

**Figure 6:**
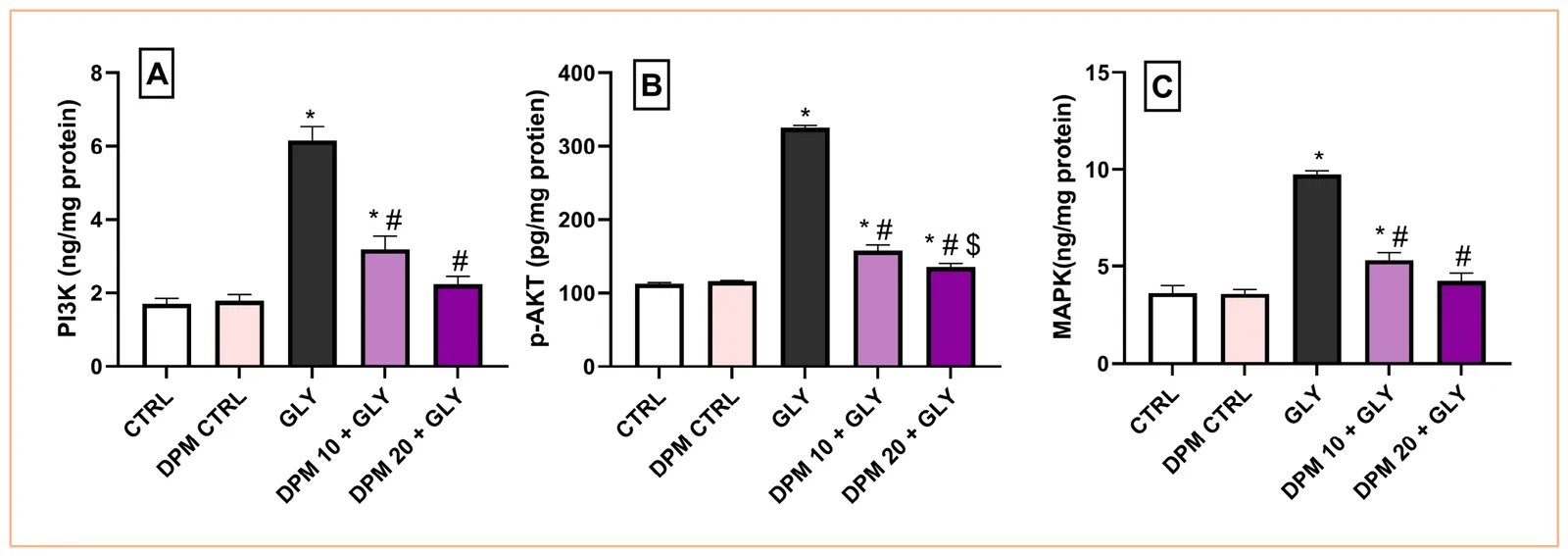
Impact of DPM (10 and 20 mg/kg) on PI3K (**A**), p-AKT (**B**), and MAPK (**C**). Values are expressed as mean ± SEM. Data were analyzed by one-way ANOVA followed by a Tukey–Kramer multiple comparison test, n = 5 (limited ELISA kit availability). * # $ *p* < 0.05 compared with CTRL, GLY, and DPM 10 + GLY, respectively. CTRL: control; DPM: dipyridamole; GLY: glycerol; MAPK: mitogen-activated protein kinase; p-AKT: phosphorylated protein kinase B; PI3K: Phosphoinositide 3-Kinase.

As shown in Figure 6, MAPK levels were significantly elevated in the GLY group, compared with the control group. Treatment with DPM (20 mg/kg) significantly reduced GLY-induced MAPK elevation, compared to the GLY group (*p* < 0.05). In a parallel pattern, PI3K/p-AKT levels were markedly elevated in the GLY group compared with control. DPM treatment attenuated both PI3K and p-AKT levels in a dose-dependent manner, compared to GLY (*p* < 0.05).

### 3.6. Impact of DPM (10 and 20 mg/kg) on Kidney Protein Expression of Bax and P53

**Figure 7:**
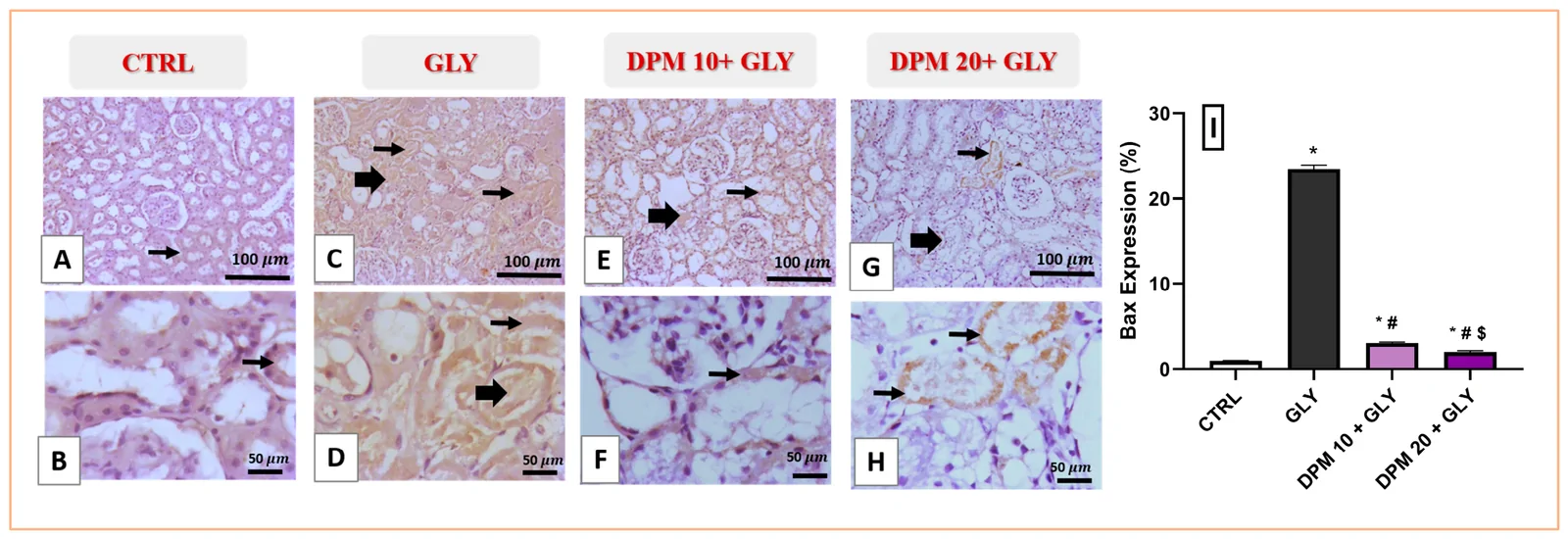
Impact of DPM (10 and 20 mg/kg) on kidney protein expression of Bax. Representative IHC images of Bax expression and localization in renal sections of different treatment groups. (**A**,**B**) CTRL group: showing negative staining or a few faintly stained cortical tubules. (**C**,**D**) GLY group: showing high immunopositive staining in renal tubular epithelium with a positively stained intraluminal cast. (**E**,**F**) DPM 10 + GLY group: showing mild, faint immunopositive cytoplasmic staining in renal tubules**.** (**G**,**H**) DPM 20 + GLY: showing focal immunopositive cytoplasmic staining in a few renal tubules. Thin arrows = positively stained renal tubules, thick arrows = positive intraluminal cast. Image magnification: 400×, bar = 50 μm, 100×, bar = 100 μm. Thick arrows indicate a positive intraluminal cast, while thin arrows indicate positively stained renal tubules. (**I**) Semi-quantification of the percentage of Bax protein expression in the groups under study. The values are given as mean ± SEM. One-way ANOVA and a Tukey–Kramer multiple comparison test were used to analyze the data (n = 6). * # $ *p* < 0.05 compared with CTRL, GLY, and DPM 10 + GLY, respectively. Bax: Bcl-2-associated X protein; CTRL: control; DPM: Dipyridamole; GLY: Glycerol.

**Figure 8:**
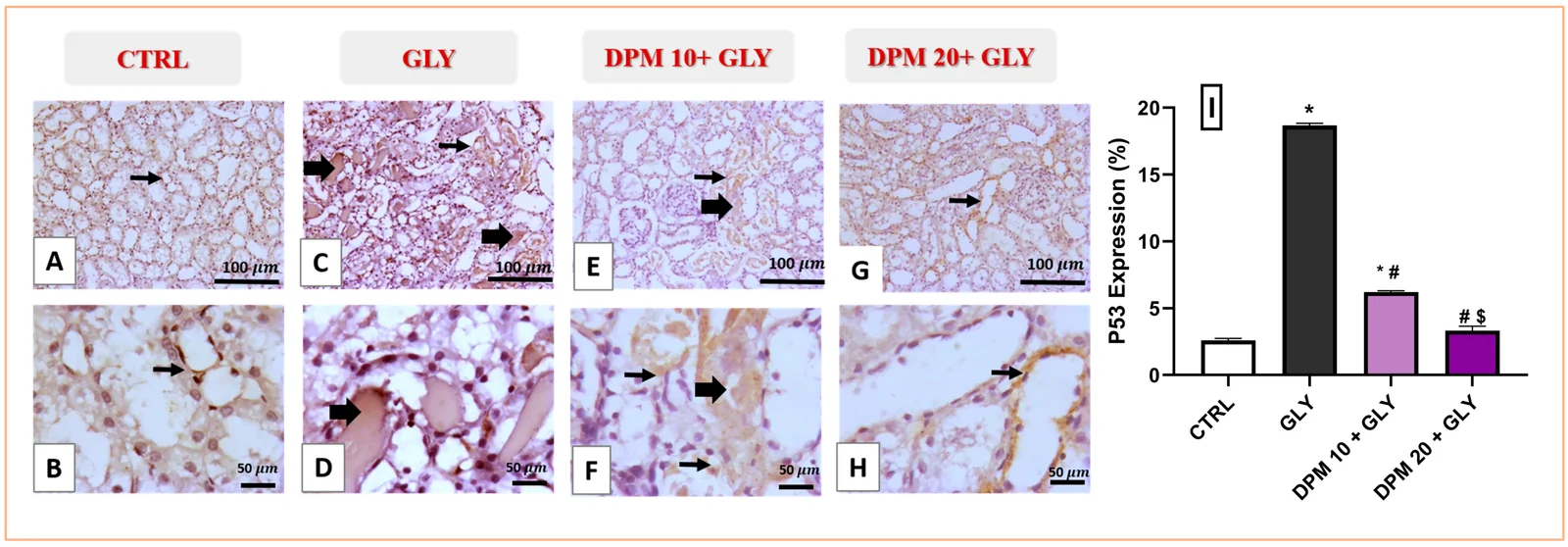
Impact of DPM (10 and 20 mg/kg) on kidney protein expression of P53. Representative IHC images of P53 protein expression and localization in renal sections of different treatment groups. (**A,B**) CTRL group: showing negative staining or a few faintly stained cortical tubules. (**C**,**D**) GLY group: showing highly immunopositive renal tubular epithelium with a positively stained intraluminal cast. (**E**,**F**) DPM 10 + GLY: showing mild, faint immunopositive cytoplasmic staining in renal tubules. (**G**,**H**) DPM 20 + GLY: showing a few areas of focal immunopositive cytoplasmic staining in renal tubules. Thin arrows = positively stained renal tubules, thick arrows = positive intraluminal cast. Image magnification: 100×, bar = 100 μm, 400×, bar = 50 μm. (**I**) Semi-quantification of the % area of P53 protein expression in the studied groups. The values are given as mean ± SEM. One-way ANOVA and a Tukey–Kramer multiple comparison test were used to analyze the data (n = 6). *, #, $ indicate *p* < 0.05 compared with CTRL, GLY, and DPM 10 + GLY, respectively. CTRL: control; DPM: dipyridamole; GLY: glycerol.

Kidney protein expression of Bax and P53 (Figure 7 and Figure 8) was significantly increased in the GLY group, compared with the control group. Treatment with DPM (10 and 20 mg/kg) significantly reduced GLY-induced Bax/P53 protein expression compared to the GLY group (*p* < 0.05).

### 3.7. Impact of DPM (10 and 20 mg/kg) on Kidney Protein Expression of Ki67 and VEGF

**Figure 9:**
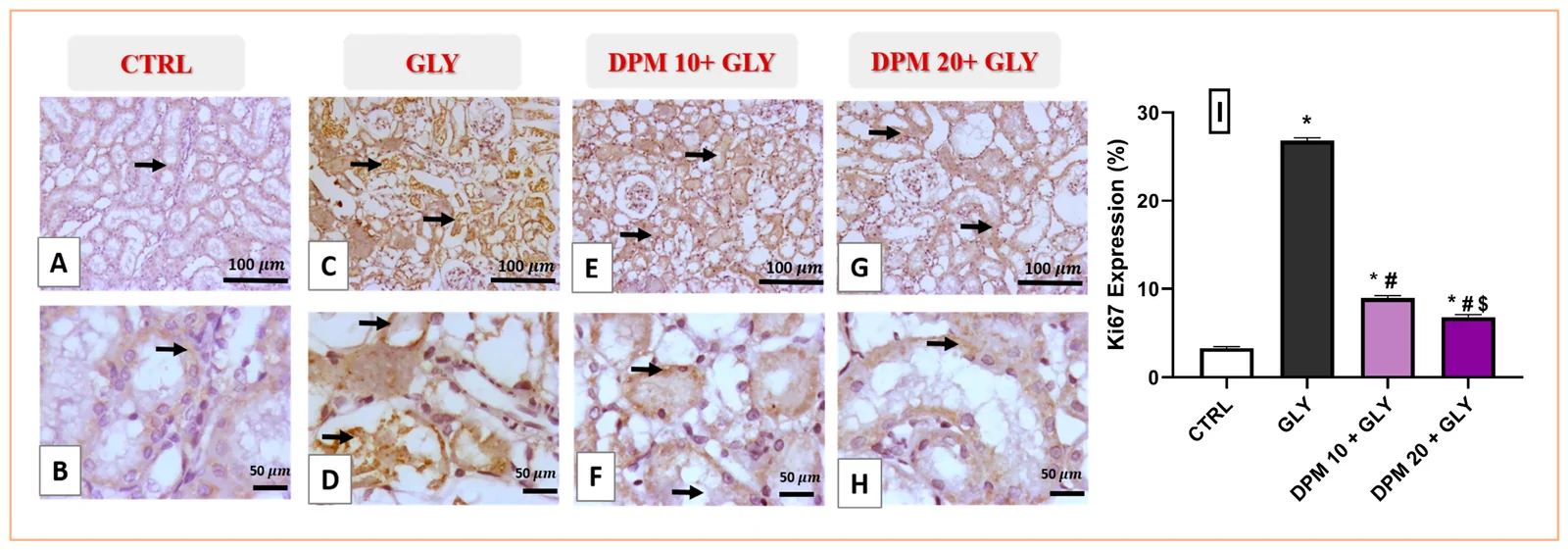
Impact of DPM (10 and 20 mg/kg) on kidney protein expression of Ki67. Representative IHC images of Ki67 expression and localization in renal sections of different treatment groups. (**A**,**B**) CTRL group: showing negative to mild, faint staining in cortical tubules. (**C**,**D**) GLY group: showing strong immunopositive staining in renal tubules with a faintly stained intraluminal cast. (**E**,**F**) DPM 10 + GLY group: showing mild-to-moderate faint immunopositive staining in renal tubules. (**G**,**H**) DPM 20 + GLY group: showing mild, faint immunopositive staining in renal tubules. Thin arrows = positively stained renal tubules. Image magnification = 100×, bar = 100 µm, 400×, bar = 50 μm. (**I**) Semi-quantification of the % area of Ki67 protein expression in the studied groups. The values are given as mean ± SEM. One-way ANOVA and a Tukey–Kramer multiple comparison test were used to analyze the data (n = 6). *, #, $ indicate *p* < 0.05 compared with CTRL, GLY, and DPM 10 + GLY, respectively. CTRL: control; DPM: dipyridamole; GLY: glycerol; Ki67: Kiel 67 (a protein strictly associated with cell proliferation).

**Figure 10:**
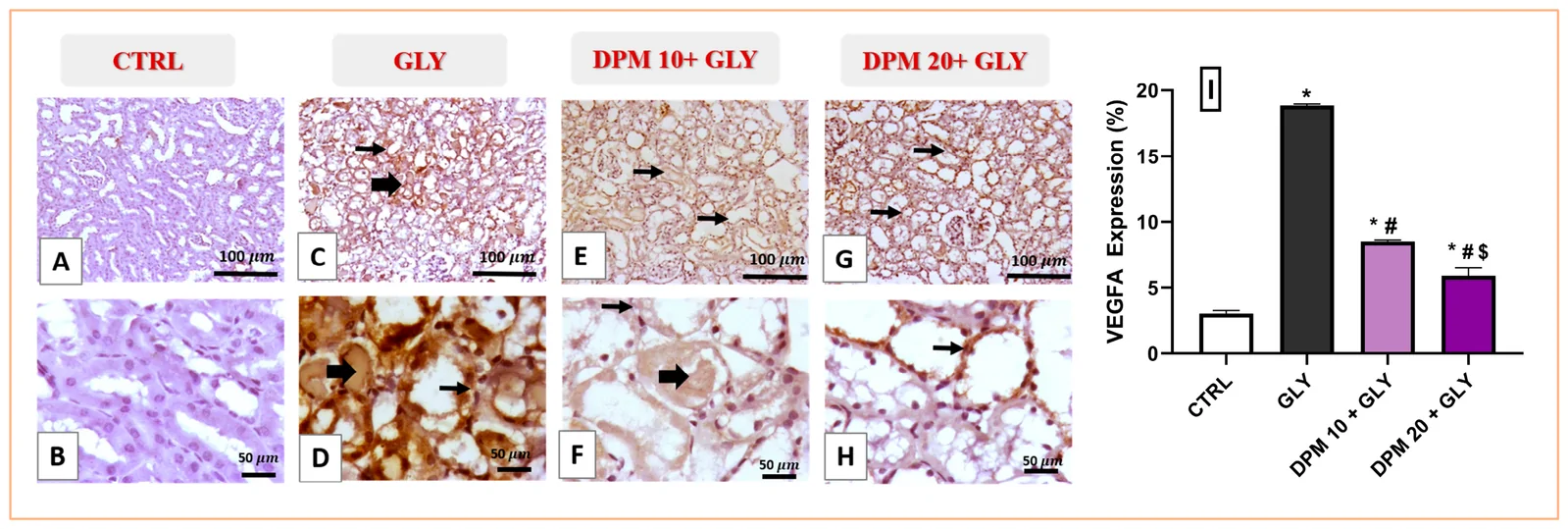
Impact of DPM (10 and 20 mg/kg) on kidney protein expression of VEGFA. Representative IHC images of VEGF expression and localization in renal sections of different treatment groups. (**A**,**B**) CTRL group: showing negative staining or a few immunopositively stained cortical tubules. (**C**,**D**) GLY group: showing high expression in renal tubules and an intraluminal cast. (**E**,**F**) DPM 10 + GLY group: showing diffuse moderate faintly immunopositive cytoplasmic staining in renal tubules. (**G**,**H**) DPM 20 + GLY group: showing mild to moderate multifocal to coalescing intensely stained renal tubules. Thin arrows = positively stained renal tubules; thick arrows = positive intraluminal cast. Image magnification = 100×, bar = 100 µm, 400×, bar = 50 µm. (**I**) Semi-quantification of the % area of VEGFA protein expression in the studied groups. The values are given as mean ± SEM. One-way ANOVA and a Tukey–Kramer multiple comparison test were used to analyze the data (n = 6). *, #, $ indicate *p* < 0.05 compared with CTRL, GLY, and DPM 10 + GLY, respectively. CTRL: control; DPM: dipyridamole; GLY: glycerol; VEGFA: vascular endothelial growth factor A.

As shown in Figure 9 and Figure 10, kidney protein expression of Ki67 and VEGF was significantly amplified in the GLY group, compared with the CTRL group. Pretreatment with DPM (10 and 20 mg/kg) significantly reduced GLY-induced elevation in Ki67 and VEGF expression, compared with the GLY group (*p* < 0.05). Interestingly, DPM’s restorative effect on Ki67 and VEGF protein expression was dose-dependent.

## 4. Discussion

The current study verified that DPM exerted significant renoprotective effects against GLY-induced RIAKI. These protective effects were reflected in improved renal function, preserved renal histological architecture, attenuated oxidative stress, reduced apoptotic markers, and enhancement of cellular survival pathways. Notably, these protective effects were dose-dependent, indicating strong pharmacological efficacy of DPM in alleviating the pathological consequences of RM-induced renal injury.

GLY-induced RM is a widely accepted experimental model that closely resembles the clinical manifestations of RIAKI. Following intramuscular glycerol injection, extensive skeletal muscle breakdown releases myoglobin, heme proteins, electrolytes, and reactive metabolites into the circulation. The excessive filtration of myoglobin through renal glomeruli promotes tubular obstruction, vasoconstriction, oxidative stress, inflammation, and tubular cell death, ultimately resulting in acute renal dysfunction [10,26].

Consistent with the aforementioned and in line with previous studies [27,28], the GLY-treated group in the present study exhibited marked elevations in serum creatinine, BUN, and CK-MB levels, indicating severe impairment of renal function and muscle injury.

DPM treatment significantly reduced serum creatinine and BUN levels, suggesting preservation of glomerular filtration and renal tubular function. Furthermore, the observed reduction in CK-MB implies attenuation of muscle injury and systemic toxicity. These beneficial effects may be explained by the multifaceted pharmacological actions of DPM, including nonselective inhibition of PDE activity and cellular adenosine uptake. This leads to increased extracellular adenosine levels, elevation of intracellular cAMP and cGMP signaling, enhancement of NO-mediated vasodilation, and improved tissue perfusion. These findings are in accordance with the cytoprotective effects of DPM in experimental and clinical models of renal injury and CKD [21,22,29].

The biochemical findings were further supported by histopathological examination, which demonstrated severe renal damage in the GLY-treated group, whereas DPM treatment markedly preserved renal architecture and attenuated tubular injury in a dose-dependent manner. These histological improvements are consistent with the observed restoration of renal function and further confirm the renoprotective efficacy of DPM.

Oxidative stress is considered a central mechanism in the pathogenesis of RIAKI. The release of myoglobin and free iron promotes the generation of ROS, causing lipid peroxidation, mitochondrial dysfunction, DNA damage, and cellular apoptosis [8,10]. In the current study, GLY administration significantly elevated renal MDA levels while reducing TAC, confirming severe oxidative stress. Increased MDA reflects enhanced lipid peroxidation of cellular membranes, whereas depletion of TAC indicates exhaustion of endogenous antioxidant defenses. These findings are consistent with previous reports demonstrating oxidative damage as a hallmark of rhabdomyolysis-associated kidney injury [8,28,30]. DPM markedly decreased MDA levels and restored TAC in a dose-dependent manner, indicating substantial antioxidant activity. This antioxidant effect may be mediated through direct scavenging of free radicals as well as activation of endogenous antioxidant pathways [31,32,33].

NO plays a complex role in renal pathophysiology. Under physiological conditions, NO maintains renal blood flow and vascular homeostasis. The marked elevation of NO levels observed following GLY administration is consistent with previous reports demonstrating induction of iNOS expression and excessive NO generation during RIAKI, leading to increased peroxynitrite formation and nitrosative tissue injury [8,34,35]. DPM significantly attenuated NO overproduction, which may be attributed to its antioxidant properties, preservation of endothelial homeostasis, and improvement of NO bioavailability through reduction of ROS-mediated NO degradation [32,33].

Mitogen-activated protein kinase (MAPK) signaling is a critical stress-responsive pathway involved in the pathogenesis of acute kidney injury. Excessive activation of MAPK cascades, particularly p38 MAPK and JNK, promotes inflammatory responses, oxidative stress, apoptosis, and tubular epithelial cell damage. The increased MAPK expression observed in the RM group is consistent with previous studies demonstrating activation of MAPK signaling during myoglobin- and GLY-induced renal injury. Activation of this pathway has been linked to enhanced production of pro-inflammatory mediators, mitochondrial dysfunction, and progression of renal tissue damage [36,37,38]. DPM significantly reduced renal MAPK expression, indicating attenuation of stress-responsive inflammatory signaling. Previous studies have demonstrated anti-inflammatory actions of dipyridamole through modulation of intracellular signaling pathways [39,40,41], although its effect on MAPK signaling in RIAKI has not been fully characterized. Collectively, these results suggest that attenuation of MAPK signaling may be one of the molecular mechanisms contributing to DPM-mediated renal protection.

GLY-provoked RM stimulates endothelin-converting enzyme-1 (ECE-1)- dependent ET-1 generation triggered by myoglobin, which drives renal vasoconstriction, a drop in renal blood flow and glomerular filtration rate, and consequent tubular damage [42]. In parallel, activation of PKC has been clearly involved in the development of RM-provoked AKI, mediating renal tubular cell damage via downstream MAPK/ERK signaling. Beyond its vasoconstrictive actions, ET-1 also activates renal cell growth, proliferation, extracellular matrix generation, inflammatory signaling, and PI3K/Akt activation. PKC and ET-1 receptor engagement may therefore represent a pathological, injury-driving arm of this pathway rather than a protective survival response in this context, leading to persistent vasoconstriction, maladaptive proliferative signaling, and tubular injury [42,43].

Although PI3K/Akt signaling is generally considered a pro-survival pathway, sustained activation under conditions of persistent oxidative stress and ET-1 overproduction may contribute to maladaptive renal remodeling and tubular injury. A myriad of studies have demonstrated the impact of the PI3K/Akt pathway on AKI. In general, as stated earlier, the PI3K/Akt pathway is mainly a pro-survival pathway; however, its role in AKI is complex, as it can play a pro-inflammatory role. In a study by Jin et al., 2022, PI3Kγ (class I group, which has a central role in the chemotactic function of macrophages and neutrophils) displayed less IRI in the kidney with fewer tubular apoptotic cells in a model of ischemia–reperfusion-induced kidney injury, proving that PI3Kγ inhibition may serve as a potential therapeutic strategy for the prevention of AKI [44].

In another study by Zheng et al., 2026, they showed that sepsis-induced AKI can upregulate the PI3K/AKT pathway and that allicin (their investigational drug) alleviated AKI by inhibiting pyroptosis via negative regulation of the PI3K/AKT pathway [45]. To further confirm their finding, they used IGF-1 (as a PI3K/AKT agonist) with allicin; the finding of this experiment showed that adding IGF-1 reversed allicin’s inhibitory effects on p-PI3K/p-AKT and pyroptosis, causing worsening of kidney function in comparison with the allicin-only group. This was carried out in vivo (CLP- or LPS-induced sepsis) and in vitro (HK-2 cells) [45].

An additional study by Ouyang et al. 2023 was carried out in vitro (human HK-2 cells), in vivo in rats with AKI induced through the ischemia–reperfusion method, and on human urine [46]. This group found that a certain circular RNA was highly expressed in human urine and in the AKI kidneys and that overexpression of that circular RNA is also associated with activation of the PI3K/AKT signaling pathway [46]. These studies suggest that downregulating the PI3K/Akt signaling pathway could have a putative role in alleviating AKI.

In our study, DPM pretreatment meaningfully and dose-dependently lessened the GLY-provoked rise in the PKC/ET-1/PI3K/Akt signaling axis. This action is consistent with the documented effects of DPM, which inhibits cellular adenosine reuptake and phosphodiesterase activity, thus increasing intracellular cAMP and cGMP levels and stimulating PKA/PKG-dependent inhibition of proliferative and kinase-driven signaling cascades [41,47]. Via these mechanisms, DPM may diminish PKC stimulation and its downstream signaling to ET-1 and PI3K/Akt signaling, besides improving renal microvascular perfusion through its vasodilatory and antiplatelet effects. Collectively, these outcomes suggest that DPM exerts its renoprotective actions, at least in part, through modulating the PKC/ET-1/PI3K/Akt signaling. It is noteworthy that downregulating PI3k/Akt pathway may have occurred due to upstream regulation of markers of injury, protecting the kidneys, and might not be due to DPM’s direct effect on PI3K/Akt. Notably, these findings exclude the association between DPM’s renoprotective effect and its modulation of this pathway; specifically, studies using specific inhibitors or gene-silencing approaches, such as siRNA, were not included in our design. This point has been added as a limitation in the current study.

Apoptosis is a major mechanism contributing to renal tubular cell loss during AKI. Among the key regulators of the intrinsic apoptotic pathway, Bax, a pro-apoptotic member of the Bcl-2 family, promotes mitochondrial outer membrane permeabilization, cytochrome c release, and subsequent activation of caspase-dependent cell death pathways. Increased Bax expression has been consistently associated with the progression of tubular injury and renal dysfunction in experimental AKI models [48,49]. Likewise, P53 serves as a critical regulator of apoptosis and DNA damage responses. Previous studies have shown substantial increases in Bax and P53 expression during GLY-induced AKI [50]. Consistent with these findings, the present study demonstrated marked increases in Bax and P53 immunoexpression in the RM group, indicating activation of apoptotic pathways. DPM treatment significantly reduced Bax and P53 expression, suggesting attenuation of apoptotic signaling and preservation of tubular cell viability. This anti-apoptotic effect may be attributed to reduced oxidative stress and modulation of PI3K/Akt signaling, both of which are known to suppress mitochondrial apoptotic pathways [51,52,53]. Furthermore, the reduction in Bax and P53 expression was consistent with the histopathological findings demonstrating decreased tubular degeneration and necrosis in DPM-treated animals at both investigated doses (10 and 20 mg/kg).

Cellular proliferation and tissue regeneration are critical determinants of renal recovery following acute kidney injury [54]. Ki67 is a well-established marker of cell proliferation and has been extensively used to evaluate renal tubular regeneration following injury [54]. Moreover, VEGFA plays a complex, context-dependent role in AKI [55,56,57], contributing to the regulation of renal microvascular integrity, endothelial survival, and tissue repair. Therefore, VEGFA was assessed in the present study to explore whether modulation of this vascular repair-related factor could contribute to the renoprotective effects of DPM. The marked elevation in Ki67 and VEGFA expression observed in the GLY-treated group may reflect an injury-associated proliferative and angiogenic response to acute tubular injury and hypoxia, rather than necessarily representing a fully regulated regenerative process [58]. DPM treatment significantly reduced both Ki67 and VEGFA expression compared with the RM group. This finding is consistent with the well-documented antiproliferative properties of DPM [47]. This antiproliferative effect has also been demonstrated directly in renal-associated cells, where DPM suppressed PDGF-stimulated proliferation of peritoneal mesothelial cells [59]. Accordingly, the reduction in Ki67 and VEGF expression following DPM treatment may reflect attenuation of excessive injury-driven proliferative and angiogenic signaling rather than impairment of tissue repair. Instead, these findings may indicate restoration of a more controlled and physiological pattern of cellular turnover, consistent with the observed improvement in renal function and preservation of renal histoarchitecture. However, Ki67 and VEGFA were only assessed at one early time point, 24 h after GLY-induced injury. While previous experimental [20] and clinical [60] investigations have shown persistent renoprotective effects of DPM in long-term treatment or follow-up, its effects on the temporal kinetics of tubular regeneration and microvascular recovery are not yet sufficiently delineated. Consequently, the present findings do not rule out a potential influence of DPM on subsequent phases of tissue regeneration. Further extended studies are essential to determine whether the initial structural protection provided by DPM correlates with sustained or improved physiological renal regeneration.

Collectively, the present findings suggest that DPM protects against GLY-induced RM-associated AKI through multiple complementary mechanisms. These include attenuation of oxidative stress and modulation of PKC, ET-1, PI3K, p-Akt, and MAPK signaling pathways. Additionally, DPM suppressed apoptosis, as evidenced by the downregulation of Bax and P53 and attenuation of Ki67 and VEGFA protein expression. Notably, the observed reductions in PI3K/AKT, Ki67, and VEGFA may reflect reduced injury-associated signaling rather than impaired renal repair, particularly given the single 24-h time point. Overall, the dose-dependent improvements observed across biochemical, molecular, immunohistochemical, and histopathological parameters support the potential of DPM as a promising renoprotective agent against rhabdomyolysis-induced AKI.

## 5. Limitations and Future Prospects

One limitation of the present study is the assessment of Ki67 and VEGFA only at one early time point (24 h after GLY-induced injury). Thus, the present findings cannot define subsequent kinetics of tubular regeneration or of microvascular recovery. Further extended studies with longer follow-up are needed to determine if DPM influences later stages of renal repair and long-term recovery. Another limitation is the need for further studies using specific inhibitors or gene-silencing approaches targeting the PI3K/AKT pathway would provide more definitive mechanistic confirmation. Additionally, the effect of DPM (10 mg/kg) alone was not studied, reflecting an incomplete factorial design as a limitation of the study.

## Figures and Tables

**Table 1:** Semi-quantitative histopathological scoring system used for the assessment of renal tissue injury.

Score	Tubular Degeneration	Tubular Necrosis	Fibrosis	Inflammation
0	None	None	None	None
1	Minimal, <10% of tubules affected; few casts	Rare, 10% of tubules affected	Minimal, <10% of interstitial area	Few, rare, <10% of examined area
2	Mild to moderate, 10–50% of tubules affected; moderate intraluminal cast	Moderate, 10–50% of tubules affected	Mild to moderate, 10–50% of interstitial area	Mild, focal, 10–50% of examined area
3	Diffuse, >50% of tubules affected; many to abundant intraluminal cast	Diffuse, >50% of tubules affected	Diffuse, severe, >50% of interstitial area	Moderate to severe coalescing, >50% of examined area

## Data Availability

The original contributions presented in this study are included in the article and Supplementary Materials. Further inquiries can be directed to the corresponding author.

## Supplementary Materials

The following supporting information can be downloaded at https://www.mdpi.com/article/10.3390/jox16060199/s1, File S1: Microscopic Original Images.

## List of Abbreviations

The following abbreviations are used in this manuscript:

AKI	Acute kidney injury
AKT	Protein kinase B
ANOVA	Analysis of Variance
Bax	Bcl-2-associated X protein
BUN	Blood urea nitrogen
cAMP	Cyclic adenosine monophosphate
cGMP	Cyclic guanosine monophosphate
CK-MB	Creatine kinase-MB
CKD	Chronic kidney disease
DPM	Dipyridamole
ECE-1	Endothelin-converting enzyme-1
ELISA	Enzyme-linked immunosorbent assay
ERK	Extracellular signal-regulated kinase
ET-1	Endothelin-1
GLY	Glycerol
H&E	Hematoxylin and eosin
IHC	Immunohistochemistry
i.m.	Intramuscular
i.p.	Intraperitoneal
IRI	Ischemia/reperfusion injury
JNK	c-Jun N-terminal kinase
MAPK	Mitogen-activated protein kinase(s)
MDA	Malondialdehyde
NO	Nitric oxide
PBS	Phosphate-buffered saline
PDGF	Platelet-derived growth factor
PI3K	Phosphoinositide 3-kinase
PKA	Protein kinase A
PKG	Protein kinase G
PKC	Protein kinase C
RIAKI	Rhabdomyolysis-induced acute kidney injury
RM	Rhabdomyolysis
SEM	Standard error of the mean
ROS	Reactive oxygen species
TAC	Total antioxidant capacity
VEGFA	Vascular endothelial growth factor A
